# Supplementary material for: SpliceVault predicts the precise nature of variant-associated mis-splicing
Source: Nat Genet. 2023 Feb 6;55(2):324–32. doi: 10.1038/s41588-022-01293-8 (PMC9925382; doi:10.1038/s41588-022-01293-8)
Supplement: Supplementary file 1 — Reporting Summary [file 41588_2022_1293_MOESM1_ESM.pdf]

nature portfolio

Corresponding author(s): Sandra T. Cooper

Last updated by author(s): Nov 29, 2022

Reporting Summary

Nature Portfolio wishes to improve the reproducibility of the work that we publish. This form provides structure for consistency and transparency in reporting. For further information on Nature Portfolio policies, see our Editorial Policies and the Editorial Policy Checklist.

Statistics

For all statistical analyses, confirm that the following items are present in the figure legend, table legend, main text, or Methods section.

n/a	Confirmed	
☐	☒	The exact sample size (*n*) for each experimental group/condition, given as a discrete number and unit of measurement
☒	☐	A statement on whether measurements were taken from distinct samples or whether the same sample was measured repeatedly
☐	☒	The statistical test(s) used AND whether they are one- or two-sided<br>*Only common tests should be described solely by name; describe more complex techniques in the Methods section.*
☒	☐	A description of all covariates tested
☒	☐	A description of any assumptions or corrections, such as tests of normality and adjustment for multiple comparisons
☐	☒	A full description of the statistical parameters including central tendency (e.g. means) or other basic estimates (e.g. regression coefficient) AND variation (e.g. standard deviation) or associated estimates of uncertainty (e.g. confidence intervals)
☐	☒	For null hypothesis testing, the test statistic (e.g. F, t, r) with confidence intervals, effect sizes, degrees of freedom and P value noted<br>*Give P values as exact values whenever suitable.*
☒	☐	For Bayesian analysis, information on the choice of priors and Markov chain Monte Carlo settings
☒	☐	For hierarchical and complex designs, identification of the appropriate level for tests and full reporting of outcomes
☐	☒	Estimates of effect sizes (e.g. Cohen's *d*, Pearson's *r*), indicating how they were calculated

Our web collection on statistics for biologists contains articles on many of the points above.

Software and code

Policy information about availability of computer code

Data collection	Custom R code was used to create 300K-RNA and is available at https://github.com/kidsneuro-lab/300K-RNA. Code developed in R version 3.6. Packages used: tidyverse 1.3.2, data.table 1.14.2. Code used to create SpliceVault is available at https://github.com/kidsneuro-lab/SpliceVault/. Code developed in R version 4.2.1. Packages used: data.table v1.14.2, DT v1.26, rintrojs v0.3.2, shinyBS v0.61.1, shinycssloaders v1.0.0, shinydashboard v0.7.2, shinyjs v2.1.0, shinyWidgets v0.7.4, DBI v1.1.3, tidyverse v1.3.2, DBI v1.1.3, odbc v1.3.3, faq v0.1.1, scales v1.1.1. Code used for SpliceAI delta score retrieval API is available at https://gitlab.com/kidsneuro/SpliceAIAPI
Data analysis	Custom R code was used to perform data analysis relevant to the paper and is available in the following repository; https://github.com/kidsneuro-lab/SpliceVault_figures. Code developed in R version 4.2.1. Packages used: data.table v1.14.2, tidyverse v1.3.2, UpSetR v1.4.0, cowplot v1.1.1, ggsci v2.9, ggpubr v0.4.0, scales v1.2.1, metR v0.12.0, readxl v1.4.1, stringr v1.4.1, jsonlite v1.8.2, TxDb.Hsapiens.UCSC.hg38.knownGene v3.15.0, openxlsx v4.2.5.1

For manuscripts utilizing custom algorithms or software that are central to the research but not yet described in published literature, software must be made available to editors and reviewers. We strongly encourage code deposition in a community repository (e.g. GitHub). See the Nature Portfolio guidelines for submitting code & software for further information.

Data

Policy information about availability of data

All manuscripts must include a data availability statement. This statement should provide the following information, where applicable:

- Accession codes, unique identifiers, or web links for publicly available datasets
- A description of any restrictions on data availability
- For clinical datasets or third party data, please ensure that the statement adheres to our policy

Source files (i.e. SRA and GTEx splice junctions processed in recount3) were downloaded from snaptron; http://snaptron.cs.jhu.edu/data/srav3h/junctions.bgz and http://snaptron.cs.jhu.edu/data/gtexv2/junctions.bgz respectively. 300K-RNA can be easily accessed and queried through SpliceVault (https://kidsneuro.shinyapps.io/splicevault/). The data used for the analyses described in this manuscript were obtained from the GTEx portal and dbGaP accession number phs000424.v8.p2 and phs000424.v9.

Human research participants

Policy information about studies involving human research participants and Sex and Gender in Research.

Reporting on sex and gender	Not relevant to this study. This was a retrospective analysis where all conceivable splice outcomes were investigated for each variant. Sex was only relevant to ascertainment "segregates with disease" criteria detailed in Bournazos et al. Sex and gender were not reported for this study.
Population characteristics	There were no covariate-relevant population characteristics of the human research participants as the experimental results of RNA diagnostic studies was not compared between patients. Variant-induced mis-splicing events were established relative to age and sex matched controls for each patient independently.
Recruitment	Ascertainment criteria detailed in Bournazos et al. Ascertain variants with high clinical suspicion of causality were (1) a high likelihood of a monogenic Mendelian disorder, (2) variant allele frequency consistent with disease incidence, (3) putative splicing variant in a clinician-defined, phenotypically concordant gene, and (4) preferably the variant segregates with disease .
Ethics oversight	Consent for diagnostic genomic testing was supported by governance infrastructure of the relevant local ethics committees of the participating Australian Public Health Local Area Health Districts. Kids Neuroscience Centre's biobanking and functional genomics human ethics protocol was approved by the Sydney Children's Hospitals Network Human Research Ethics Committee (protocol 10/CHW/45 renewed with protocol 2019/ETH11736 (July 2019 – 2024)) with informed, written consent for all participants.

Note that full information on the approval of the study protocol must also be provided in the manuscript.

Field-specific reporting

Please select the one below that is the best fit for your research. If you are not sure, read the appropriate sections before making your selection.

☒ Life sciences ☐ Behavioural & social sciences ☐ Ecological, evolutionary & environmental sciences

For a reference copy of the document with all sections, see nature.com/documents/nr-reporting-summary-flat.pdf

Life sciences study design

All studies must disclose on these points even when the disclosure is negative.

Sample size	Determined by the number of variants for which we had experimental evidence to show were splice-altering through our RNA diagnostics program (Bournazos et al).
Data exclusions	Variants that did not alter splicing and variants creating or modifying the essential splice site motif of a cryptic splice site were excluded.
Replication	All splicing outcomes were experimentally confirmed by at least 2 primer pairs as per our standardized RNA diagnostics practices outlined in Bounazos et al. Some variants were confirmed by both RNAseq and RT-PCR.
Randomization	We did not randomise in our analysis of genetic splice-altering variants. All splicing variants were included in this study for which robust RNA assay data was available and met our inclusion criteria. Ascertainment criteria detailed in Bournazos et al. Ascertain variants with high clinical suspicion of causality were (1) a high likelihood of a monogenic Mendelian disorder, (2) variant allele frequency consistent with disease incidence, (3) putative splicing variant in a clinician-defined, phenotypically concordant gene, and (4) preferably the variant segregates with disease . For certain analyses we took random samples of RNA-seq samples (i.e. figure 2E and extended data figure 9).
Blinding	N/A. The was a retrospective analysis were all conceivable splicing outcomes were investigated for each variant.

Reporting for specific materials, systems and methods

We require information from authors about some types of materials, experimental systems and methods used in many studies. Here, indicate whether each material, system or method listed is relevant to your study. If you are not sure if a list item applies to your research, read the appropriate section before selecting a response.

Materials & experimental systems

n/a	Involved in the study
☒	☐ Antibodies
☒	☐ Eukaryotic cell lines
☒	☐ Palaeontology and archaeology
☒	☐ Animals and other organisms
☒	☐ Clinical data
☒	☐ Dual use research of concern

Methods

n/a	Involved in the study
☒	☐ ChIP-seq
☒	☐ Flow cytometry
☒	☐ MRI-based neuroimaging
