# Supplementary figures and images for: SpliceVault predicts the precise nature of variant-associated mis-splicing
Source: Nat Genet. 2023 Feb 6;55(2):324–32. doi: 10.1038/s41588-022-01293-8 (PMC9925382; doi:10.1038/s41588-022-01293-8)

OPHN1

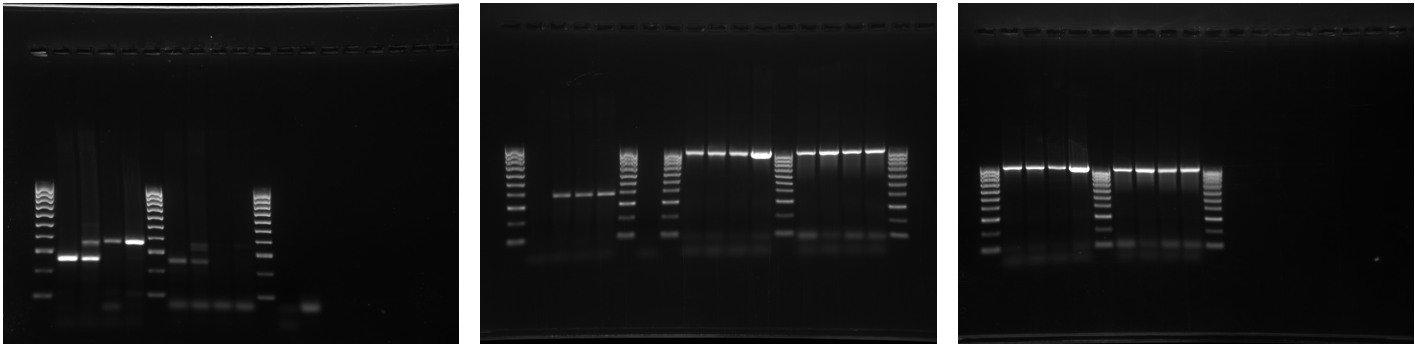

SPG11

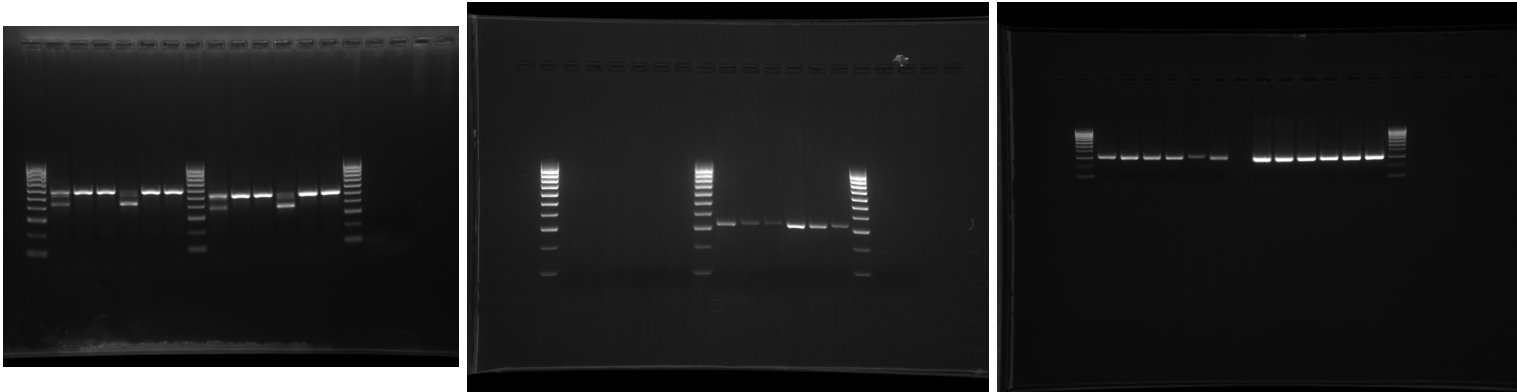

GSDME

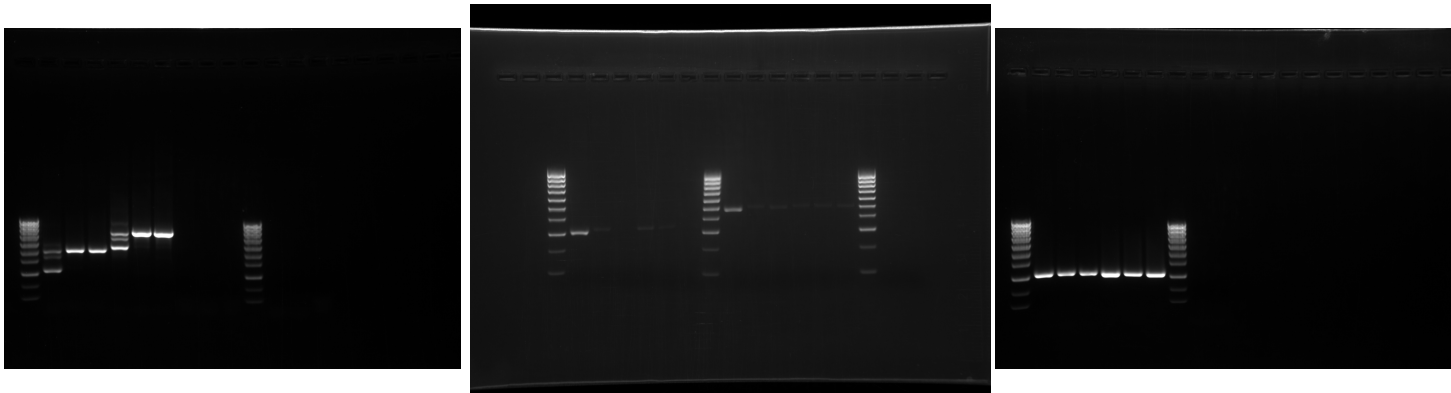

EMD

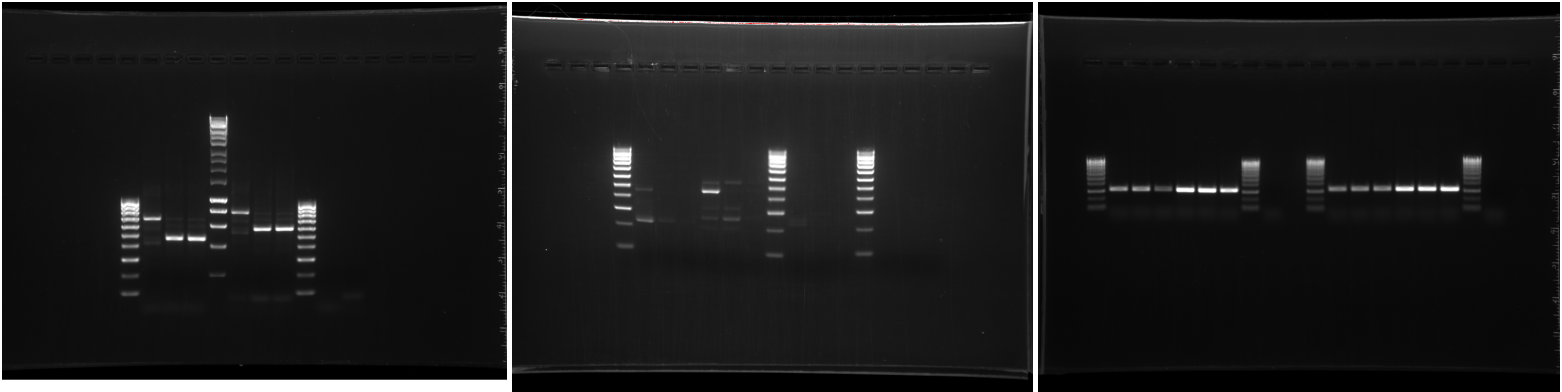

Supplement: Source Data Extended Data Fig. 8 — Unprocessed gels. [file 41588_2022_1293_MOESM15_ESM.pdf]
